# Naturopaths practice behaviour: provision and access to information on complementary and alternative medicines

**DOI:** 10.1186/1472-6882-5-15

**Published:** 2005-07-11

**Authors:** Caroline Smith, Karen Martin, Elizabeth Hotham, Susan Semple, Geraldine Bloustien, Deepa Rao

**Affiliations:** 1 School of Health Sciences, University of South Australia, North Terrace, Adelaide, South Australia, Australia; 2 School of Pharmaceutical Molecular and Biomedical Sciences, University of South Australia, North Terrace, Adelaide, South Australia, Australia; 3 School of Communication Information and New Media, University of South Australia, St Bernards Road, Magill, South Australia, Australia

## Abstract

**Background:**

The increasing use of complementary and alternative medicines in Australia has generated concern regarding the information on these products available to both healthcare providers and the public. The aim of this study was to examine the practice behaviours of naturopaths in relation to both the provision of and access to information on complementary and alternative medicines (CAM).

**Methods:**

A representative sample of 300 practicing naturopaths located nationally were sent a comprehensive survey which gathered data on self reported practice behaviour in relation to the provision of information on oral CAM to clients and the information needs of the practitioners themselves

**Results:**

A response rate of 35% was achieved. Most practitioners (98%) have a dispensary within their clinic and the majority of practitioners perform the dispensing themselves. Practitioners reported they provided information to clients, usually in the form of verbal information (96%), handwritten notes (83%) and printed information (75%). The majority of practitioners (over 75%) reported always giving information on the full name of the product, reason for prescribing, expected response, possible interactions and contraindications and actions of the product. Information resources most often used by practitioners included professional newsletters, seminars run by manufacturers, patient feedback and personal observation of patients. Most practitioners were positive about the information they could access but felt that more information was required in areas such as adverse reactions and safe use of CAM in children, pregnancy and breastfeeding. Most naturopaths (over 96%) were informed about adverse events through manufacturer or distributor newsletters. The barriers in the provision of information to clients were misleading or incorrect information in the media, time constraints, information overload and complex language used in printed information. The main barrier to the practitioner in information access was seen as the perceived division between orthodox and complementary medicine practitioners.

**Conclusion:**

Our data suggest most naturopaths were concerned about possible interaction between pharmaceuticals and CAM, and explore this area with their patients. There is scope to improve practitioners' access to information of adverse events including an increased awareness of sources of information such as the Australian Therapeutic Goods Administration (TGA) website.

## Background

The use of complementary medicines in Australia has become commonplace. In 2002 it was estimated that 52% of the Australian population had used at least one non-physician-prescribed complementary medicine in the previous year[1]. These medicines are widely available from many sources including health food stores, supermarkets, direct marketing, natural therapy clinics and pharmacies. Although self-prescription of oral complementary medicines is common, research has also shown an increase in visits to alternative practitioners, in particular naturopaths and herbal therapists[1]. Both of these types of practitioners commonly prescribe oral complementary medicines.

The Expert Committee on Complementary Medicines in the Health System in Australia was convened in 2003 and asked to consider the regulatory, health system and industry structures necessary to ensure that the objectives of the National Medicines Policy (NMP) and in particular that of the National Strategy for Quality Use of Medicines (QUM), were met in relation to complementary medicines. The report from the Expert Committee raised a number of concerns surrounding the information available to healthcare providers and consumers regarding complementary medicines, and one of the recommendations of the committee was the commissioning of a study to determine the needs of healthcare professionals and consumers on complementary medicines and the options available for conveying this information[2].

Previous research has surveyed the naturopathic and Western herbalists workforce[3], providing an overview of the types of therapies and diagnostic procedures used by these therapists, the form and labelling of complementary medicine preparations used in the practice, cost of treatment to the client, practitioner income, and adverse events experienced within the practice. Whilst this survey found that nutritional medicines were generally provided as over-the-counter commercial formulations, the methods for preparing and dispensing herbal and homoeopathic medicines varied, with one half of practitioners mixing or combining 90% or more of their herbal medicines in their own clinics. The practitioners in this survey also reported a substantial number of adverse events associated with these medicines. However this research did not address the practice behaviours of these practitioners in regard to the provision of information to clients on the complementary medicines which were prescribed at the consultation or information on complementary medicines accessed by the practitioner themselves.

Research in the USA has also examined practice patterns of naturopathic physicians[4]. The demographics of these practitioners were found to be remarkably similar to those of naturopaths in Australia. American naturopathic physicians commonly prescribed oral complementary medicines, with the most common of these being botanical medicines, vitamins, minerals and homoeopathic preparations. Again, this study did not address questions relating to the provision of or access to information on oral complementary medicines by naturopathic practitioners.

In Australia the profession of naturopathy is essentially unregulated. A level of self-regulation is exerted by the professional associations in the form of minimum qualification levels and monitoring of annual requirements such as Continuing Professional Education, however there are a large number of these associations and entry requirements vary between the groups. Several Australian States are currently exploring options for the regulation of naturopaths and other complementary medicine practitioners [5].

The current agreed minimum qualification level enforced by the main professional associations is Advanced Diploma in Naturopathy. This qualification is contained within the Health Training Package HLT02 and represents a consistent base level of training within the industry. The Advanced Diploma courses are taught at privately-owned Vocational Education and Training Colleges which are registered through the various State Education Authorities. Some of these colleges are also registered to deliver degree courses, and several universities also deliver courses in naturopathy.

The purpose of this study was to examine the practice behaviours of naturopaths in relation to the provision of and access to information on oral complementary medicines. In particular we were interested to know what are their counseling and advice-giving behaviors and are these behaviors adequate to ensure safe and judicious use of oral complementary medicines. The study had two aims:

1. to explore the information provided by naturopaths to clients on these medicines, circumstances under which information would not be provided to a client, and the types of questions clients ask in respect to oral complementary medicines, and

2. to examine the skill base of naturopaths with accessing information, how practitioners find out about adverse events and changes in regulation to complementary medicines and their confidence in respect to answering client questions about these medicines.

## Methods

We designed a short self completion questionnaire with the aim of collecting data from naturopaths describing their practice behaviors, education and training, information gathered during a consultation with a client, experience with accessing information and knowledge about complementary and alternative therapies and socio demographic characteristics. The questionnaire adopted and adapted questionnaires that had been used to assess practice behaviors and practitioner knowledge around CAM and the use of other over the counter medicines [6-9]. A total of 36 questions were included with the inclusion of some open ended questions to examine access to information on CAM, any barriers to the provision of information on complementary medicines and suggestions for reducing any perceived barriers. The questionnaire was piloted with a small group of naturopaths in South Australia and minor revisions made. The questionnaire took approximately 20 minutes to complete. The study was approved by the Human Research Ethics Committee at the University of South Australia.

The sampling frame was based on a population of 3,000 naturopaths from three States in Australia. The sample size was estimated at 300, based on an estimate that 90% of naturopaths would actively prescribe herbs, with a 95% confidence interval, this allows for a 5% error and a 50% response rate. A stratified sample was dawn from three States in Australia.

Data were collected from a national survey of practicing naturopaths in Australia. A representative sample of 300 naturopaths was undertaken from a listing of naturopaths held by the Medical Benefits Fund (MBF), a large national health insurance fund in Australia. It was decided to sample from a database held by a health fund because of the absence of a central database of naturopaths held by any professional association. MBF did not release the list of practitioners but undertook the sampling procedure under direction of the research team and provided the administrative support to mail out the questionnaires. The Fund generated a list of active naturopaths defined as those who have had a claim paid in the previous 12 months. Subjects were assigned a random number selected by a researcher independent from the study team.

Naturopaths were sent a questionnaire with a covering letter explaining the purpose of the study and a reply paid envelope. Two reminders were sent out to facilitate the return of questionnaires. Statistical analysis was performed using SPSS version 11.5 (Chicago, IL, USA)[10]. Frequencies and percentages and other descriptive statistics were used to describe the data.

## Results

In total 300 questionnaires were sent out, and 110 were returned. However, five of these were non practicing practitioners giving a response rate of 35%. The majority identified themselves as Caucasian and female and were in the 45–54 age category. A diploma was the standard qualification (Table 1). Naturopaths received training in the modalities of Western herbal medicine (96%), nutritional supplementation (94%), diet therapy (90%), remedial therapies (78%) and homoeopathy (74%). Over a third of practitioners worked full time in practice, working for 29 (+/- 14.5 SD) hours in clinic per week and over half of practitioners (51%) had been in practice for less than 10 years. Where demographic data was available age, gender and education were compared with data from the naturopaths and Western herbalists workforce survey[3]. The demographics appear consistent with 76% of practitioners being female with a mean age of 44 years.

**Table 1:** Socio-demographic characteristics of Naturopaths

Socio demographic characteristics of naturopaths			Demographic data from naturopathic workforce survey***	
	N	%	N = 795	%
Currently practicing as a naturopath	105	95.5		
Gender				
Male	31	30.0	191	24.0
Female	73	70.0	604	76.0
Age			44.0	10.4
18–34 years	20	19.0		
35–44	29	27.6		
45–54	34	32.4		
55+	21	20.0		
Educational level**			3.1 years	
Diploma	49	46.7		
Advanced diploma	47	44.8		
Degree	36	34.3		
Higher degree	9	8.6		
Ethnic background				
Caucasian	100	96.2		
Asian	3	2.9		
Other	1	1.0		

*Mean and SD, ** Not mutually exclusive percentages do not add up to 100% because a practitioner may hold more than one qualification.

** *Data from Bensoussan A, Myers SP, Wu SM, O'Connor K (2004)

### Practice behaviour

Most practitioners (103, 98%) reported having a dispensary in their clinic, with 101 (97%) naturopaths performing the dispensing themselves, and a small number (7%) using unqualified staff. Seventy-eight percent of naturopaths always advised their patients to purchase their products from their clinic, with the remainder advising their patients to purchase their products from the pharmacy or health food shop.

Twenty eight (27% of the sample) never dispensed a repeat medication before undertaking a follow up consultation, while 74 (71%) practitioners often or sometimes did so. Generally, there was a reluctance to dispense medications without a consultation. Practitioners reported never dispensing a product without full consultation: (1) if the patient was presenting with a different condition (45% of practitioners), (2) if the patient had consulted with another naturopath (49%), and (3) if the patient requested it (58%).

### Counselling, screening and advice giving behaviour

The majority of naturopaths (96%) gave verbal information to their patients on complementary medicines. Most also provided written information which included practitioner handwritten notes (83%), printed information (75%) and less often printed information from journal articles (26%), text books (25%) or manufacturer information (35%).

The content of information presented to patients varied between practitioners (Table 2). Information on dose was given by almost all practitioners. Over 75% of practitioners always gave information on the full name, reason for prescribing the product, possible interactions, expected response, contra-indications, and the action of the product. Seventy five percent of naturopaths did not 'always' present information relating to safe use, for example information on possible adverse effects, interaction with other substances, names and ingredients and information on effectiveness. Lack of time was given as the main factor why individual ingredients were not discussed with the patient. All naturopaths asked new patients about their use of orthodox Western medicine, and over 90% of naturopaths always asked about the use of Western medicines when they recommended or dispensed CAM or were concerned about a potential interaction.

**Table 2:** Information given to patients about prescribed products

	Always		Sometimes		Rarely		Never		Missing	
	N	%	N	%	N	%	N	%	N	%
Recommended dose	101	96.2	1	1.0					3	2.9
Full name	91	86.7	11	10.5					3	2.9
Reason for prescribing	89	84.8	6	5.7	1	1.0			9	8.6
Possible interaction with medication	85	81.0	14	13.3	4	3.8			2	1.9
Medical condition requiring caution	84	80.0	17	16.2	3	2.9			1	1.0
Expected response	84	80.0	121	10.5	4	3.8	2	1.9	4	3.8
Product actions	82	78.1	17	16.2	3	2.9	1	1.0	2	1.9
Possible adverse reactions	78	74.3	20	19.0	2	1.9	1	1.0	4	3.8
Interaction with other substances	62	59.0	30	28.6	11	10.5			2	1.9
Names of ingredients	54	51.4	40	38.1	8	7.6	2	1.9	21	1.0
Evidence of effectiveness	22	21.0	52	49.5	23	21.9	8	7.6		

### Confidence in their own knowledge

The approach in counselling on appropriate use of naturopathic products will be influenced by the practitioner's training and their skills in life long learning. Ninety one (83%) naturopaths reported their formal training met their needs when performing the day to day practice of naturopathy.

There are a number of information resources available to practitioners and we asked naturopaths to indicate the frequency with which they used a particular resource. The resources used most *often *by practitioners to obtain information on CAM included articles in professional newsletters (91%), reference textbooks (72%), continuing professional education (CPE) seminars run by manufacturers (70%), patient feedback (66%), personal observation of patient response (58%), and CPE activity run by other industry bodies (55%). Use of the scientific literature or discussion with other health professionals was reported less often. For example the resources used *sometimes *included discussion with naturopathic colleagues (49%), journal articles describing a case study (43%), journal articles describing randomised clinical trials (41%), information from course notes (43%), popular health magazines (44%) and reference web sites (37%). Information sources used *rarely *by practitioners included interaction with pharmacists (46% of naturopaths), medical doctors (44% of naturopaths) or other health care practitioner (40% of naturopaths).

Naturopaths' views on the adequacy of information resources they can draw on to help answer questions relating to CAM are reported in Table 3. The majority of naturopaths (76%) reported the available resources describing the benefits of CAM were very adequate. Over 50% of naturopaths identified five areas of practice where information resources were not very adequate. Consequently, in response to another question, at least 40% of practitioners responded not feeling very confident in answering questions relating to these five areas namely 1- use of CAM during pregnancy and when breastfeeding, 2- adverse effects, 3- safety in relation to medical conditions, 4- questions about interactions between CAM and other medicines and 5- questions on the regulatory status of CAM.

**Table 3:** Views on the adequacy of information resources to answer questions about CAM

	Very adequate		Somewhat adequate		Not adequate		Not applicable	
	n	%	n	%	n	%	n	%
Benefits of CAM	78	75.7	22	21.4	2	1.9	1	1.0
Safety of CAM re children	59	57.3	36	35.0	8	7.8		
Safety of CAM re pregnancy and breastfeeding	54	48.5	45	43.7	8	7.8		
Adverse effects of CAM	51	49.0	42	40.4	9	8.7	1	1.0
Safety of CAM re medical conditions	45	43.3	43	41.3	16	15.4	1	0.9
Interactions of CAM and other medicines	33	31.4	53	50.5	17	16.2	2	2.0

A number of factors were expressed as being very important in influencing practitioner confidence with providing information to their patients. This included: knowledge of these products (95%), access to scientific or clinical information (85%), belief in the effectiveness of these products (84%), belief in the quality of the products (84%) and belief in the safety of the products (81%).

### Information awareness regarding safety of CAM

A safety concern relating to the use of CAM are adverse events associated with herbal medicines. Over 96% of naturopaths reported they were notified about adverse events through manufacturer or distributor newsletters. Other sources of notification were from professional associations (88%) and professional association journals, website or email discussion lists (86%), CPE (69%) and informal discussion with colleagues (60%). Less than 30% of the naturopaths used the Therapeutic Goods Administration (TGA) (the Australian regulatory authority for therapeutic goods) website as a source of notification of adverse events.

Practitioners were asked how their knowledge of adverse events could be improved. Fifty percent of practitioners identified formal education as a need and 58% identified improved access to CPE activities and other relevant information sources were needed. Formal training or improved skills to assess quality scientific or clinical evidence was viewed positively by 30% of naturopaths.

Practitioners were asked open ended questions regarding whether the information they were able to access about CAMs was adequate, what they considered the barriers to the provision of information about CAMs to patients were, and what suggestions they had for ways of overcoming these barriers.

The majority of practitioners were very positive about the information they could access although there were common themes in these answers regarding the time taken to access quality information. A number of practitioners mentioned the need for unbiased information and expressed concerns regarding the objectivity of the information that was supplied by the manufacturers of those products.

In general, practitioners expressed strong opinions about what they perceived to be the barriers to their own access to information. The perceived division between orthodox and complementary medicine practitioners was the strongest theme presented. Themes identified less frequently were perceived Government bias against complementary medicines, the cost of accessing information and the lack of research. Other comments related to the need for professional registration of naturopaths and a need for orthodox medical practitioners to have a greater awareness of and training in complementary medicines.

With regard to the information provision to patients, the barriers were seen as misleading or incorrect information in the media, time constraints within the consultation, information overload on the part of the client, information supplied with the product in language too complex for the client, and self-prescribing of CAMs through over-the-counter sales of these products. When suggesting ways to overcome these barriers, practitioners sought greater recognition and status of the naturopathic profession and increased integration and communication with the orthodox health community. Many practitioners also felt that restricting CAM products from over-the-counter sales and allowing them to be supplied by complementary practitioners only would assist with information provision to the client. There were also calls for manufacturers to include more information on their products and for greater balance in media reporting of CAM-related issues.

## Discussion

Self reported data from this study reported on the practice behaviour of naturopaths in relation to providing information on complementary medicines. Our data suggest most naturopaths are concerned about safety in relation to possible interaction between orthodox Western medicine and CAM and this is an area naturopaths explore with their patients during the consultation. There is scope to improve the practice behaviour of some naturopaths to ensure greater safety by giving consideration to reducing the number of practitioners who would dispense a product without consultation (on solely the patient's request), and not using unqualified staff to dispense a product.

We found both consistencies and inconsistencies between practitioners where information on the product was presented. Most practitioners reported providing information on the dose, the full name, reason for prescribing the product, possible interactions, expected response, contra-indications, expected response and the action of the product. However, information was not provided by 40% or more of practitioners on possible adverse effects, interaction with other substances, and the names and ingredients of the herbal product. A bias resulting from the self report of behaviour can not be excluded.

With an increasing profile of safe use of complementary medicines, improved labelling by the naturopath has the potential to reduce public health concerns and increase the judicious use of herbal medicines. Although the majority of prescribing took place in the clinic, 22% of practitioners advised their patients to purchase product from the pharmacy or health food store. Patients who are sent to these outlets may or may not be given a written directive with the item to be purchased. Patients may be sold product which is different to that recommended by the naturopath, be given conflicting advice regarding dosage or may simply forget the instructions given to them, particularly when those instructions differ from that printed on the label.

Initial naturopathic training met the needs of naturopaths with performing the day to day practice of naturopathy. We have identified a number of deficiencies that will need to be addressed to ensure naturopaths can remain up to date with increasing quality information on CAM and continue to provide high standards of counselling. Naturopaths identified that information resources relating to the safety of CAM use in children, use during pregnancy and breastfeeding, the adverse effects of CAM, the safety of using CAM as related to certain medical conditions and interactions of CAM and other medicines as not very adequate. Given the insufficient research base describing any adverse effects in some of these patient groups, providing evidence based practice in these areas will remain inadequate and naturopaths will need to continue practising with caution.

Naturopaths use a wide variety of information sources to obtain information on CAM, although conventional health care practitioners and practitioners from another CAM discipline were not widely used as a resource to access information. This is similar to the findings from the workforce survey where practitioners reported that they felt well prepared for practice within their clinical training with the exception of the area of inter-professional communications[3]. Our findings also suggest that practitioners do not rely heavily on information sources such as scientific literature that are most likely to provide quality evidence on the use or non use of complementary medicines. A notable proportion of practitioners identified a need for continuing professional education in this area and further development of skills with assessing quality scientific or clinical evidence may lead to greater reliance on evidence based information sources.

The naturopathic and Western herbal medicine workforce survey reported that adverse events were relatively common, and calculated that naturopaths would on an average encounter 1.2 adverse events in their patients per year of full time practice[3], or one event each 11 months of practice[2]. This indicates that the access to quality information related to adverse events associated with oral CAMs is critical to the safe use of these medicines. The Governments' response to *Complementary Medicines in the Australian Health System *(2003) identified a need to improve access to information about adverse reports[11]. Our study also highlighted the need for a greater awareness of the Australian regulatory TGA website within the practitioner population. There is also a need for the positive promotion of the TGA as an information source, as the 'anti-CAM' perception of the Government held by some of the practitioners may contribute to these practitioners not accessing Government based information sources. Practitioners also identified that their knowledge of adverse events could be improved.

A limitation of this study is the low response rate which may limit the generalisations we can make relating to the national population of naturopaths. The demographic characteristics of our study population allow limited comparison with published data from national surveys from Australia and the United States[2,3]. Data from the two surveys in Australia are comparable with respect to gender and age ranges of naturopaths. The questions asked on education can not be compared directly, however three years training in naturopathy in Australia would equate to the award of a diploma. Data from the USA survey indicates the majority of practitioners are Caucasian and this is comparable to data in our survey. The similarities between the two studies suggest the results may be generalisable to a wider population of naturopaths in Australia. The self reported questions may give rise to a bias and this highlights a need for observational research of practice behaviour to confirm our findings.

## Conclusion

The findings from this study are timely in relation to the Australian Government's recognition of the need to identify the information and skills needed by health care professionals to assess the quality of evidence concerning the use of complementary medicines. This study provides baseline data for describing the practice behaviors in naturopaths in relation to counseling and advice-giving behaviors and their skills in accessing information on CAM adverse events. It will facilitate the evaluation of practice behaviour over time. The majority of naturopaths in Australia report behaviours that suggest they provide appropriate and judicious use of oral CAMs for their patients but the practitioners identify the need for further training to ensure safe and judicious practice continues in the future.

## Competing interests

The author(s) declare that they have no competing interests.

## Authors' contributions

CS conceptualised the research, analysed the data and took the lead with writing the paper.

KM participated in conceptualising the research, study design, analysed the data and contributed to the preparation of the manuscript.

EH participated in the study design and reviewed the manuscript.

SS participated in the study design and reviewed the manuscript.

GB participated in the study design and reviewed the manuscript.

DR participated in the study design and reviewed the manuscript.

## Pre-publication history

The pre-publication history for this paper can be accessed here:


